# A 3.2 kW Single Stage Narrow Linewidth Fiber Amplifier Emitting at 1050 nm

**DOI:** 10.3390/mi15070871

**Published:** 2024-06-30

**Authors:** Xiaoxi Liu, Xin Tian, Binyu Rao, Baolai Yang, Xiaoming Xi, Zefeng Wang

**Affiliations:** 1 College of Advanced Interdisciplinary Studies, National University of Defense Technology, Changsha 410073, China; 2 Nanhu Laser Laboratory, National University of Defense Technology, Changsha 410073, China; 3 State Key Laboratory of Pulsed Power Laser Technology, Changsha 410073, China

**Keywords:** fiber laser, MOPA, narrow-linewidth, high-power

## Abstract

In this paper, we have demonstrated a narrow linewidth high power fiber laser emitting at a short wavelength of ~1050 nm. The fiber laser is based on a structure of master oscillator power amplification (MOPA) with an optimized fiber Bragg-grating-based laser cavity as the seed. Both stimulated Brillouin scattering (SBS) and stimulated Raman scattering (SRS) effects have been effectively suppressed by using a long passive fiber between the seed and the amplifier. Based on the fiber amplifier, we have ultimately boosted the narrow linewidth laser from ~40 W to 3.2 kW with a slope efficiency of 85.1% and a 3-dB linewidth of ~0.1 nm. The SRS suppression ratio of the laser is ~29.7 dB at maximum power. Due to our fiber mode control strategies, the beam quality always stays near-diffraction-limited while amplifying, and the measured M^2^ factor is ~1.4 at the maximum power. Further increase in output power is limited by the SBS effect.

## 1. Introduction

High-power fiber lasers, especially those with narrow linewidths, are necessary for applications such as spectral beam combining (SBC), nonlinear frequency conversion (NFC), coherent beam combining (CBC), and gravitational wave detection (GWD) [1,2,3,4]. In recent years, over 20 kW high-power lasers operating in conventional waveband (1070~1080 nm) have been reported by several groups [5,6,7]. Theoretically, fiber lasers operating at short waveband (<1060 nm) have the advantage of possessing a higher threshold of transverse mode instability (TMI) due to their lower quantum defect heating and a stronger gain saturation effect [8]. However, the record output power of the short wavelength fiber lasers is much lower than those in a conventional waveband because of the limitation of amplified spontaneous emission (ASE) [9]. Meanwhile, the Raman gain spectrum of these lasers overlaps with the emission spectrum of Yb3+ and results in a much stronger SRS effect [10,11]. It is even more difficult to achieve high-power output when dealing with narrow linewidth fiber lasers, since stimulated Brillion scattering (SBS) [12,13] should be considered as well.

Recently, many high-power narrow linewidth fiber lasers below 1060 nm have been reported. In 2016, Naderi et al. demonstrated a 1 kW laser operating at 1034 nm with a 3 dB linewidth of 0.011 nm based on a phase-modulated single-frequency laser (PMSFL) seed [14]. In 2020, Chu Q et al. realized a 3 kW monolithic narrow linewidth fiber amplifier at 1030 nm, which achieved a 0.18 nm spectrum linewidth, with the OSNR being 37 dB and near-diffraction-limited beam quality on the premise of a theoretical model [15]. In 2021, Xu Y et al. suppressed SRS by optimizing the length of the Yb-doped fiber and the pumping scheme, achieving a 2.4 kW narrow-spectral-width near-diffraction-limited monolithic fiber laser system at ~1045.2 nm in a fiber Bragg grating (FBG)-based master oscillator power amplifier (MOPA) configuration with a signal-to-noise ratio of ~33 dB [16]. In 2022, Zheng Y et al. studied a pump-sharing oscillator-amplifier (PSOA) structure theoretically and experimentally, and submitted a 1050 nm fiber laser that delivers 3.1 kW with the linewidth being 0.22 nm at 3 dB, the beam quality maintaining at M^2^~1.33, and the optical signal-to-noise ratio (OSNR) being 45.5 dB based on the PSOA structure [17]. In 2024, Ma P et al. demonstrated a 5-kW power-level narrow-linewidth fiber amplifier at 1050 nm with a PMSFL as the seed and a homemade biconical-tapered Yb-doped fiber as the active fiber. The 3 dB linewidth is ~0.54 nm and the beam quality is M^2^~1.5 [18]. 

However, the phase-modulation is restricted by the intense SBS, complex structures, and expensive costs. Multi-mode oscillators have the advantages of simple structure, excellent stability, and high cost-effectiveness, but their output is a multi-longitudinal-mode laser with poor time-domain stability, easy spectral broadening, and susceptibility to nonlinear effects. In this letter, we established a high-power narrow-linewidth fiber laser emitting at 1050 nm based on a FBG seed and MOPA configuration. By optimizing the seed and amplifier properties, the output power reached 3.2 kW and slope efficiency attained 85.3%, with the 3 dB linewidth being ~0.1 nm and the signal-to-Raman ratio being ~29.7 dB at maximum output power. The beam quality M^2^ factor was 1.4 at an output power of 3.2 kW.

## 2. Experimental Setup

**Figure 1:**
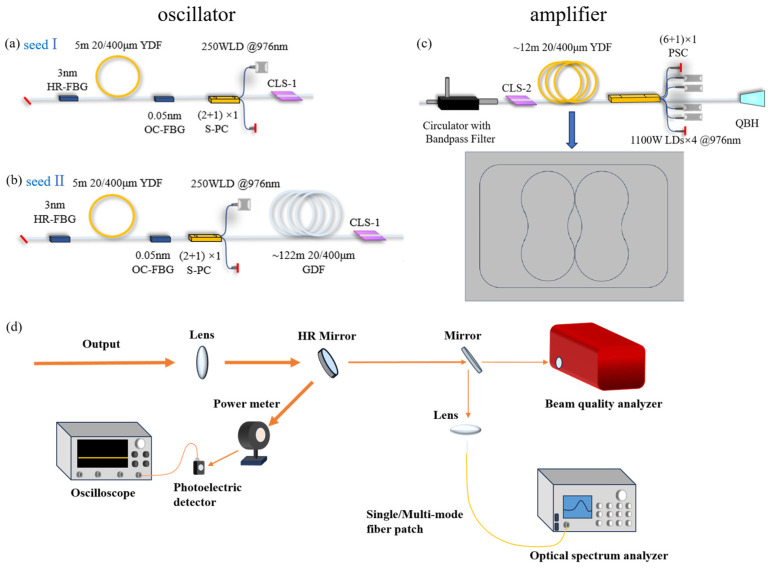
(**a**,**b**) Schematics of seed I and seed II. (**c**) Schematics of the amplifier and the YDF coiling shape. (**d**) A schematic of the measurement system.

First of all, an FBG-based fiber oscillator named seed I was established as showed in Figure 1a. The oscillator consists of a piece of active fiber and a pair of FBGs with a central wavelength of ~1050 nm. One end of the HR FBG is spliced with the Yb-doped fiber (YDF), and the other end is angle-cleaved to avoid facet Fresnel reflection. A counter-pumping scheme was adopted, and a 976 nm wavelength-stabilized LD was coupled into the linear cavity by a (2 + 1) × 1 side-pumped combiner (S-PC). The reflectivity of the high-reflectance (HR) and output coupler (OC) FBGs are 99.5% and ~9.7%, respectively. The 3 dB bandwidth of the HR and OC FBGs are 3.1 nm and 0.05 nm, respectively. A ~5 m YDF with a core/cladding diameter of 20/400 μm and an absorption coefficient of 1.3 dB/m @ 976 nm was used as the gain media. In order to effectively control the beam quality of the seed, the YDF was coiled on a water-cooled plate with a bending diameter from 8 cm to 10 cm. A cladding light stripper (CLS-1) was spliced to the output fiber of the S-PC to filter out the cladding light, thus protecting fiber components after the seed.

In the case of seed II, as displayed in Figure 1b, a piece of passive fiber (GDF) with a core/cladding diameter of 20/400 μm and a length of ~122 m was inserted between the S-PC and the CLS-1 in the experiment. The laser properties of the two seeds were characterized, and their power boosting ability were compared by using the same high-power fiber amplifier. 

The amplifier stage was based on a counter-pumping configuration, as shown in Figure 1c. A hybrid fiber component combining a band-pass filter (BPF) and a circulator was employed between the seed and the amplifier. On the one hand, the spectral noise of the seed can be filtered and the SRS suppression ratio can be enhanced by the BPF. On the other hand, the backward signal can be isolated by the circulator and the SBS power can be monitored at the backward port of the circulator. Another cladding light stripper (CLS-2) was applied to protect the circulator from the residual pump power of the amplifier. A (6 + 1) × 1 backward pump/signal combiner was connected to four groups of 976 nm wavelength-stabilized LDs with an output power of 1100 W for each. The input and output signal fibers of the combiner have a core/cladding diameter of 25/400 µm and 25/250 µm, respectively. Another piece of YDF with the same parameters as the ones used in the seed was applied as the gain fiber in the amplification stage. 

In order to increase the bending loss of high-order modes (HOMs) and thus suppress the TMI effect, the 12 m-long YDF was coiled on a double-eight-shaped water-cooled aluminum plate (as illustrated in Figure 1d) with the minimum/maximum diameter of ~8 cm and ~10.5 cm, respectively. The water-cooling temperature maintains 20 °C for the pump sources and the fiber amplifier. A homemade quartz block head (QBH) attached with a CPS was spliced after the fiber amplifier with the core and cladding diameter of 50/400 µm.

The output laser was characterized with a setup shown in Figure 1d. The collimated laser was divided by a high-reflective mirror (HR Mirror). More than 99% of the power was reflected to the power meter. Part of the scattered light from the sensor was collected using a photoelectric detector to analyze the temporal characteristics (Tektronix, MSO64, Beaverton, OR, USA). After the HR mirror, the transmitted light was equally divided with a 50:50 beam splitter. One part of the light was coupled into fiber patch cords to measure the spectrum using an optical spectrum analyzer (OSA, Yokogawa 6370D, Tokyo, Japan). The other part was received by a beam quality analyzer (SP920, Ophir Photonics) to measure the M^2^ factor. 

## 3. Experimental Results and Discussions

### 3.1. In the Case of Seed I

**Figure 2:**
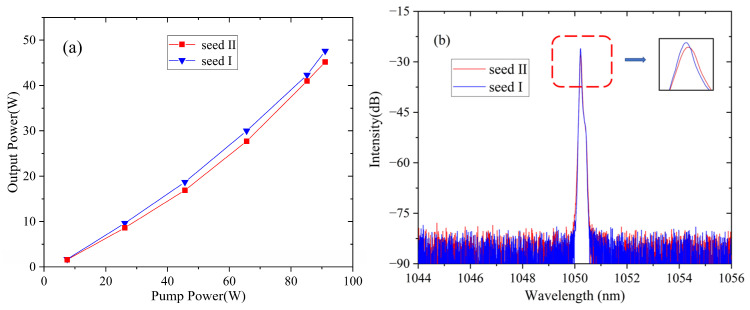
(**a**) The output power of seed I and seed II versus pump power. (**b**) Spectra of seed I and seed II when the output power is around 42 W. Inserted: zoom in of the spectral peak region.

First of all, the output laser properties of seed I and seed II were characterized and are shown in Figure 2. The output power, as shown in Figure 2a, increases linearly with the pump, and the slope efficiency is ~54.6% at the maximum of ~44.2 W. The measured beam quality was M^2^~1.3. When the two seeds operated at the same pumping power, the output spectra of the seed are displayed in Figure 2b. In the case of seed II, the output power reduced to 42.6 W, with a slope efficiency of 52.6% due to the background loss of the attached GDF. When the long GDF was deposited, the FWHM of the spectrum was also broadened from 0.042 nm to 0.062 nm because of the nonlinear effect (such as self-phase modulation) in the long passive fiber.

**Figure 3:**
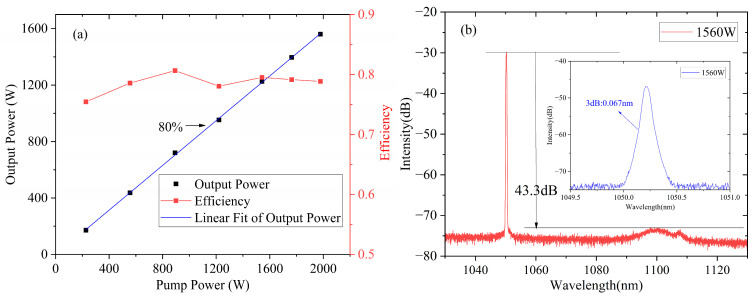
The results of MOPA under the situation of seed I. (**a**) Output power and O-O efficiency under a different pump; (**b**) Spectrum at the maximum output.

Then, these seeds were connected to the amplifier successively. In the case of seed I, the forward output power and the O-O efficiency versus pump power are illustrated in Figure 3a. The output spectrum of the laser is shown in Figure 3b.

As shown in Figure 3a, the forward power increases linearly with the pump power, with a slope efficiency of ~80%. The measured output spectrum at the maximum power of ~1560 W (Figure 3b) represents the narrow linewidth laser, which has a high SNR with a SRS suppression ratio of ~43.3 dB. The 3 dB linewidth was broadened from ~0.042 nm to 0.067 nm during the power amplification, with a spectral broadening ratio of 20.8 pm/kW. However, the backward power increased exponentially when the output power exceeded ~1500 W. In order to prevent damage of the laser, the achieved maximum output power was 1560 W, and further power boosting was limited by the SBS effect.

### 3.2. In the Case of Seed II

**Figure 4:**
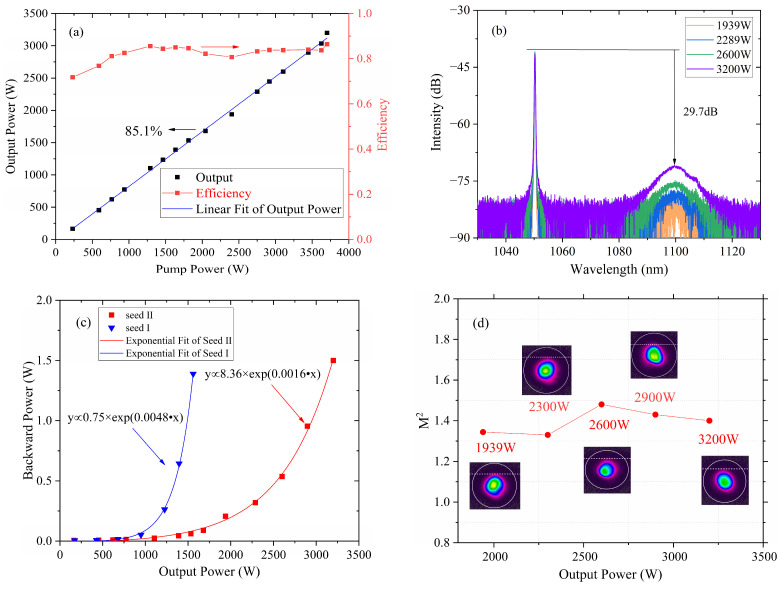
Output characteristics of the amplifier in the case of seed II. (**a**) Output power and O-O efficiency. (**b**) Spectrum measured with a multi-mode patch cord. (**c**) Backward power evolution in the case of the two seeds. (**d**) Measured beam quality under different output powers. Inserts: beam profiles at their beam waists.

In order to enhance the SBS threshold and further boost the output power of the amplifier, a long piece of GDF was inserted between the oscillator and the amplifier. The output power of seed II was set to almost the same power level as seed I, and the spectrum was shown in Figure 2b. Figure 4a displays the output power and optical efficiency versus pump power under the new structure. It is perceived that the output power has achieved 3.2 kW when the total pumping power reached 3.703 kW, with a slope efficiency of approximately 85.1%. It can be seen that there is no roll-off in the output power, indicating that the amplifier is free of TMI. 

As illustrated in Figure 4b, the output spectrum was collected with a multi-mode fiber patch cord and measured with an OSA. It shows that the Stokes component of the SRS at ~1100 nm gradually increases and reaches 29.7 dB at the highest power. The backward powers of the two seeds were also measured at the third port of the circulator. As presented in Figure 4c, both of them increased exponentially and reached ~1.5 W at output powers of 1.5 kW and 3.2 kW, respectively. The SBS effect was suppressed enormously, and the SBS threshold of seed II was remarkably twice that of seed I. As shown in Figure 4d, the beam quality of the output laser was also measured by a beam quality analyzer. During the laser amplification process, there was no evidence of the occurrence of the TMI effect, and the M^2^ factor remains at ~1.4.

**Figure 5:**
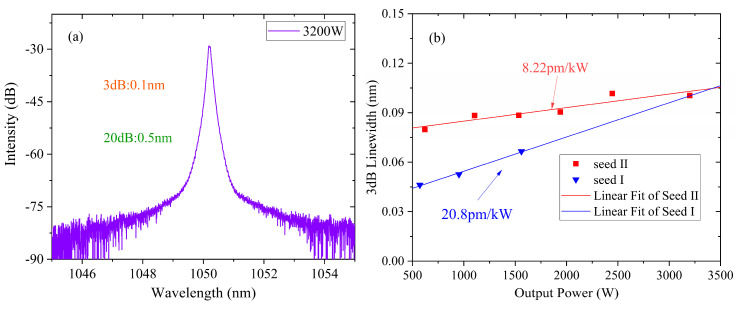
(**a**) Output spectrum at the maximum power in the case of seed II. (**b**) Spectral broadening tendency of the amplifier.

To precisely evaluate the linewidth of the laser, a single-mode fiber patch cord was used to collected the signal, and the results are shown in Figure 5. At the maximum output power, the 3 dB and 20 dB linewidth are ~0.10 nm and ~0.50 nm, respectively. Figure 5b shows the tendency of linewidth with pump raising. These curves exhibit that the 3 dB linewidth was broadened from 0.067 nm to 0.088 nm at a power of 1534 W when seed II was applied. It is obvious that the when the GDF was involved, the highest output power of 3200 W was achieved, and the 3-dB linewidth was maintained at 0.10 nm at maximum power. Seed II provides a spectral broadening rate of 8.22 pm/kW, which is less than half of the rate of seed I. The linear fit of the 3 dB linewidth indicates that these two seeds might have the same linewidth if they were both boosted to 3.2 kW. To the best of our knowledge, this is the narrowest linewidth achieved by a fiber amplifier with an output power more than 3 kW based on a narrow linewidth FBG-based seed. Further increases in power are limited by the pump power. 

It is worth noting that the shortcoming of the FBG-based narrow linewidth seed is the instability in the time domain. Plenty of self-locked pulses generated in the cavity cause an instantaneous increase of the signal laser and then significantly increases energy density in the fiber optic cores, resulting in decreases in the thresholds of nonlinear effects, such as SPM, four-wavelength mixing (FWM), and SRS [19,20,21]. Thus, strong spectral broadening and the SRS effect are easily generated during the process of amplification. 

**Figure 6:**
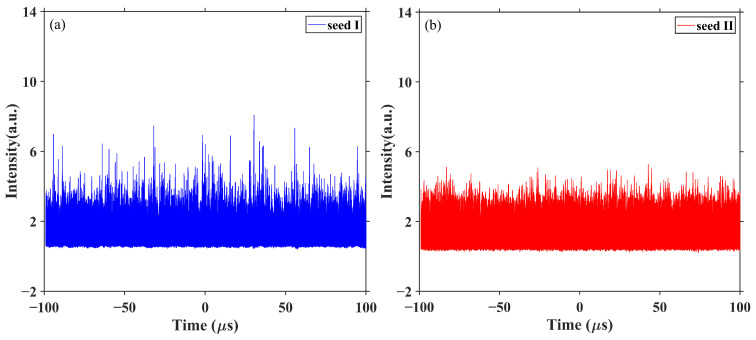
The comparison results of temporal stability in the case of (**a**) seed I and (**b**) seed II.

On the one hand, the output spectrum is broadened while passing through the long piece of GDF, and results in the enhancement of the SBS threshold. On the other hand, an increase in length of the resonant cavity causes Self-Phase Modulation (SPM), which causes the division of pulses and the weaking of temporal peak intensities. Therefore, the nonlinear effect suppression can be attributed to the spectrum broadening and the temporal stabilization due to the long passive fiber. This is also confirmed by the measured temporal traces of the seeds as shown in Figure 6. 

**Figure 7:**
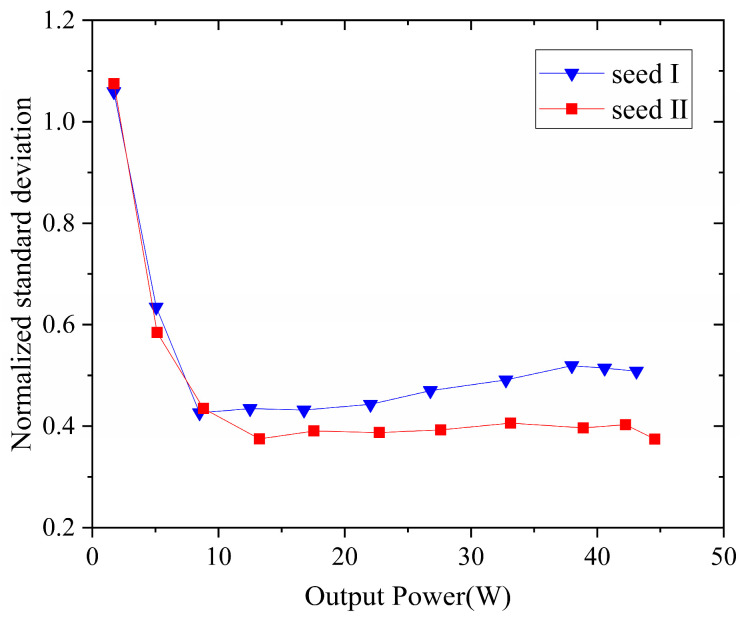
Normalization standard deviation of temporal data of seed I and seed II.

The calculated normalized standard deviation (NSTD) of the time traces is plotted in Figure 7. It is obvious that the NSTD decreases at the beginning and then gradually approaches a stable value versus the pump. In the case of seed I, the NSTD dropped from 1.06 to 0.47 and then remained stable when the output power exceeded 10 W. This value goes to 0.37 with an improvement of ~21.3% in the case of seed II. Therefore, the experimental results indicate that the amplifier exhibits a lower spectrum broadening rate and a higher SBS threshold in the case of seed II. We attribute this to the improvement of the temporal properties of the seed.

## 4. Conclusions

In this paper, we experimentally established a narrow linewidth high power fiber amplifier with an FBG-based fiber oscillator as the seed. The temporal properties of the seed operating at 1050 nm was improved by attaching a long piece of GDF at the output and the SBS threshold of the amplifier increased from 1560 W to >3000 W. Finally, based on the improved seed, a maximum output power of 3.2 kW was achieved with the O-O efficiency of 85.1%. At maximum output power, the beam quality M^2^ factor is ~1.4, and the Raman suppression ratio is 29.7 dB. The 3 dB linewidth and 20 dB linewidth are ~0.10 nm and ~0.50 nm, respectively. To the best of our knowledge, this is the reported highest output power and narrowest linewidth in this type of NLFA. Further increases in output power are limited by the SBS effect. 

## Data Availability

Data underlying the results presented in this paper are not publicly available at this time but may be obtained from the authors upon reasonable request.

## References

[1] Jiang M., Ma P., Huang L., Xu J., Zhou P., Gu X. (2017). kW-level, narrow-linewidth linearly polarized fiber laser with excellent beam quality through compact one-stage amplification scheme. High Power Laser Sci. Eng..

[2] Shi W., Fang Q., Zhu X., Norwood R.A., Peyghambarian N. (2014). Fiber lasers and their applications. Appl. Opt..

[3] Kablukov S.I., Dontsova E.I., Akulov V.A., Vlasov A.A., Babin S.A. (2011). Frequency doubling of Yb-doped fiber laser to 515 nm. Laser Phys..

[4] Liu Z., Jin X., Su R., Ma P., Zhou P. (2019). Development status of high-power fiber lasers and their coherent beam combination. Sci. China Inf. Sci..

[5] Shiner B. The Impact of Fiber Laser Technology on the World Wide Material Processing Market. Proceedings of the CLEO: Science and Innovations 2013.

[6] Xi X., Yang B., Zhang H., Pan Z., Huang L., Wang P., Yang H., Shi C., Yan Z., Chen Z. (2023). 20 kW monolithic fiber amplifier directly pumped by LDs. High Power Laser Part. Beams.

[7] Xiao H., Pan Z., Chen Z., Ma P., Liu W., Yang H., Yan Z., Wang M., Xi X., Li Z. (2024). 20 kW fiber laser with high beam quality enabled by tapered ytterbium-doped fiber. High Power Laser Part. Beams.

[8] Liu Z., Ma P., Tao R., Wang X., Zhou P. (2015). Study of wavelength dependence of mode instability based on a semi-analytical model. IEEE J. Quantum Electron..

[9] Yan P., Wang X., Li D., Huang Y., Sun J., Xiao Q., Gong M. (2017). High-power 1018 nm ytterbium-doped fiber laser with output of 805 W. Opt. Lett..

[10] Chu Q., Shu Q., Chen Z., Li F., Yan D., Guo C., Lin H., Wang Z., Jing F., Tang C. (2022). Experimental study of mode distortion induced by stimulated Raman scattering in high-power fiber amplifiers. Photonics Res..

[11] Hejaz K., Shayganmanesh M., Rezaei-Nasirabad R., Roohforouz A., Azizi S., Abedinajafi A., Vatani V. (2017). Modal instability induced by stimulated Raman scattering in high-power Yb-doped fiber amplifiers (Article). Opt. Lett..

[12] Russell P.S.J., Culverhouse D., Farahi F. (1991). Theory of forward stimulated Brillouin scattering in dual-mode single-core fibers. IEEE J. Quantum Electron..

[13] Zhou P., Ma P., Liu W., Xiao H., Ren S., Song J., Xu J., Chen Y., Lai W. High power, narrow linewidth all-fiber amplifiers. Proceedings of the Conference on Fiber Lasers XIX—Technology and Systems at SPIE LASE Conference.

[14] Naderi N.A., Dajani I., Flores A. (2016). High-efficiency, kilowatt 1034 nm all-fiber amplifier operating at 11 pm linewidth. Opt. Lett..

[15] Chu Q., Shu Q., Liu Y., Tao R., Yan D., Lin J., Wang F. (2020). 3 kW high OSNR 1030 nm single-mode monolithic fiber amplifier with a 180 pm linewidth. Opt. Lett..

[16] Xu Y., Sheng Q., Wang P., Cui X., Zhao Y., Xu H., Ding X., Fang Q., Shi W., Yao J. (2021). 2.4 kW 1045 nm narrow-spectral-width monolithic single-mode CW fiber laser by using an FBG-based MOPA configuration. Appl. Opt..

[17] Zheng Y., Han Z., Li Y., Li F., Wang H., Zhu R. (2022). 3.1 kW 1050 nm narrow linewidth pumping-sharing oscillator-amplifier with an optical signal-to-noise ratio of 45.5 dB. Opt. Express..

[18] Ma P., Pan Z., Yao T., Yang H., Chen Y., Liu W., Wang X., Wang Z., Zhou P., Chen J. (2024). 5 kW power-level 1050 nm narrow-linewidth fiber amplifier enabled by biconical-tapered active fiber. Opt. Lett..

[19] Leng J., Gao Y., Guo S., Jiang Z., Wang W. (2015). Influence of temporal characteristics on the power scalability of the fiber amplifier. Laser Phys..

[20] Tsang Y.H., King T.A., Ko D.K., Lee J. (2006). Output dynamics and stabilization of a multi-mode double-clad Yb-doped silica fiber laser. Opt. Commun..

[21] Li T., Ke W., Ma Y., Sun Y., Gao Q. (2019). Suppression of stimulated Raman scattering in a high-power fiber amplifier by inserting long transmission fibers in a seed laser. J. Opt. Soc. Am. B.

